# In Situ Programming of Shape-Morphing Hydrogels via Vat Photopolymerization for 4D Bioprinting

**DOI:** 10.3390/gels12050382

**Published:** 2026-04-30

**Authors:** Luca Guida, Elisa Ciotti, Giovanni Venturelli, Simone Bagatella, Marco Cavallaro, Marinella Levi

**Affiliations:** Department of Chemistry, Materials and Chemical Engineering “Giulio Natta”, Politecnico di Milano, 20133 Milan, Italy; elisa.ciotti@mail.polimi.it (E.C.); giovanni.venturelli@polimi.it (G.V.); simone.bagatella@polimi.it (S.B.); marco.cavallaro@polimi.it (M.C.)

**Keywords:** 4D bioprinting, vat photopolymerization, crosslinking density, swelling, crosslinking gradient, finite element method

## Abstract

The fabrication of complex architectures remains a central challenge in 3D bioprinting, as the low mechanical properties of hydrogels limit the range of achievable geometries. Four-dimensional (4D) bioprinting can address these limitations by enabling programmed shape-morphing behavior; however, in most approaches, this functionality is introduced after hydrogel formation, limiting the complexity of the resulting deformation. Here, a proof-of-concept strategy is presented, in which shape-morphing is directly encoded during fabrication. By modulating light exposure time layer-by-layer in vat photopolymerization, spatial variations in crosslinking density are introduced in situ within Gelatin Methacryloyl (GelMA) hydrogel constructs. Exposure times in the range of 20–70 s were investigated, enabling controlled bending of the printed structures upon immersion in aqueous media, with radii of curvature between 11 and 20 mm depending on the geometry. This approach allows deformation pathways to be programmed during printing, without requiring additional materials or post-processing steps. The morphing behavior was further supported by finite element simulations, which reproduced the experimentally observed deformation and enabled prediction of the shape change. In addition, high cell viability (>95%) was maintained after material contact and UV exposure. Overall, this study demonstrates that swelling-driven actuation can be encoded during fabrication. Although demonstrated on simplified geometries, this approach provides a versatile framework for process-driven shape-morphing and represents a step toward more spatially resolved and potentially volumetric 4D bioprinting strategies.

## 1. Introduction

In the field of 3D bioprinting, the ability to fabricate complex three-dimensional architectures is essential for reproducing the structural and functional features of living tissues [1]. Applications such as vascular scaffolds, where branching and interconnected channels are required to sustain cell viability and tissue integration, exemplify this need [2]. Yet, such designs remain challenging to realize with hydrogels, the most widely used materials for biomedical applications [3,4]. Their intrinsic low stiffness limits the fabrication of stable, higher-order geometries [5,6].

The two most widely used techniques, extrusion-based bioprinting (EBB) and vat photopolymerization (VP), offer complementary advantages: EBB enables the deposition of a wide variety of cell-laden bioinks [7,8], while VP provides high resolution and precise control over geometry [9,10]. Despite these strengths, both approaches encounter inherent difficulties when fabricating constructs with overhangs, inclined features, or unsupported regions [11]. Indeed, when hydrogels are employed, their low mechanical strength and tendency to deform under their own weight further exacerbate these limitations, making the reliable reproduction of complex freeform structures particularly challenging [12].

Overcoming the limitations imposed by hydrogel softness and the constraints of current printing strategies requires a new approach that goes beyond static structures. One promising direction is the use of stimuli-responsive hydrogels, which can change shape or mechanical properties in a controlled manner after printing [13]. Shape-morphing hydrogels have been reported in several fields, including but not limited to tissue engineering [13], flexible electronics [14,15] and soft robotics [16]. By carefully programming these materials, it becomes possible to generate constructs that transform over time. This enables features that are difficult or impossible to print directly, such as complex folds, branching networks, or self-supporting overhangs. Moreover, this approach opens new opportunities for the development of responsive biofabricated systems capable of activating under specific stimuli, enabling controlled and site-specific release functionalities [17,18].

This concept, commonly referred to as four-dimensional (4D) bioprinting, extends traditional 3D bioprinting by adding a temporal dimension, in which the geometry of a construct evolves in response to external cues such as temperature, pH, light, or hydration [19,20]. Different classes of stimuli-responsive hydrogels have therefore been developed, each exploiting a specific trigger to induce shape changes with distinct advantages and limitations [21,22].

pH-responsive hydrogels enable controlled deformation within specific chemical environments, but the narrow physiological range limits their applicability in cell-laden constructs [23]. Light-responsive systems, often based on azobenzene [24] or spiropyran [25] chemistries, offer precise and rapid actuation and are well-suited for repeated shape changes; however, maintaining a deformed configuration typically requires prolonged or continuous irradiation, which poses challenges for cell viability and practical implementation in larger constructs [26,27]. Temperature-sensitive hydrogels can undergo reversible gel–sol transitions, but the degree of deformation is constrained by the narrow temperature window compatible with biological systems [28,29]. In contrast, hydration-induced shape changes, arising naturally from swelling, are inherently biocompatible and broadly applicable. Although swelling is often considered a limitation, it can be harnessed as a controlled actuation mechanism. In this way, an intrinsic material behavior becomes a design feature for programmable shape-morphing [19].

Many strategies have been developed to induce shape-morphing in hydrogel-based constructs, each exploiting specific material behaviors or structural designs. A common approach relies on thin hydrogel sheets combined with swelling gradients, such as differential hydration across the material thickness. For example, exposing one side of a hydrogel to moisture while limiting water access to the opposite side generates directional bending. This method takes advantage of aqueous environments as a biocompatible stimulus and can produce predictable curvatures in cell-laden constructs. However, for tissue engineering applications, where constructs must be fully immersed in culture medium, maintaining a hydration gradient is not feasible, effectively eliminating this strategy. In addition, controlling diffusion rates and achieving rapid, precise actuation in larger or volumetric constructs remains challenging, and the resulting deformations are typically restricted to simple bending [30,31,32].

Another widely adopted approach involves the fabrication of bilayered hydrogels, in which two layers with distinct swelling properties are combined. Upon immersion, the differential expansion between layers generates bending or folding toward the less swollen side. This method can produce simple curvatures, tubular structures, or self-folding sheets and is compatible with cell-laden hydrogels such as GelMA and alginate derivatives [33,34,35,36,37].

Exploiting unidirectional crosslinking gradients is a strategy chosen by several works in the literature. Gradients are achieved by incorporating a photoabsorbing compound into the hydrogel precursor and exposing the material to UV light from one direction. The absorber attenuates the light as it penetrates the thickness, creating a gradient in crosslinking density: regions closer to the light source become more crosslinked and swell less, while regions further away remain softer and swell more. Upon immersion, this differential expansion induces curvature or folding driven by internal stresses [5,26,38].

Finally, two-dimensional crosslinking patterns extend the design space further. In this approach, a patterned light source, often a digital projector, is used to selectively expose regions of a hydrogel plane, creating spatially controlled crosslinking. This enables complex planar folding patterns and localized curvature without the need for multiple material layers [38,39].

Despite the advances described above, current 4D hydrogel strategies remain limited by the way shape-morphing behavior is encoded within the construct. In most cases, the deformation is not intrinsically programmed throughout the volume, but rather imposed through simplified geometries or surface-confined crosslinking patterns. As a result, these approaches are often limited to thin sheets or simple architectures, where the morphing response is governed by uni- or bidimensional gradients rather than fully spatially resolved distributions.

This limitation largely arises from the decoupling between fabrication and functional encoding. Shape-morphing behavior is commonly introduced through post-processing steps or external stimuli, such as light, applied from a single direction. Consequently, the achievable crosslinking patterns are inherently constrained, preventing the formation of complex internal gradients across the thickness of the construct.

As a result, the range of accessible deformation modes remains restricted. For instance, configurations requiring coexisting regions with opposite curvature within the same structure, or more intricate folding pathways, are challenging to achieve using reported approaches.

The constraints highlight the need for strategies that integrate shape-morphing encoding directly during fabrication, enabling more spatially resolved and potentially volumetric control over the programmed response, while remaining compatible with established manufacturing techniques.

In this context, the key challenge is to encode differential swelling behavior directly during the printing process, rather than introducing it through material heterogeneity or post-fabrication treatments.

This work presents a 4D bioprinting approach based on vat photopolymerization, in which shape-morphing behavior is encoded directly during fabrication through layer-by-layer modulation of light exposure time. Unlike conventional strategies that rely on material composition or post-fabrication treatments, the proposed method leverages a process parameter intrinsic to the printing technique to introduce functionality without additional materials or processing steps.

By varying the exposure time across successive layers, spatial differences in crosslinking density are generated within GelMA hydrogel constructs. Upon immersion in aqueous media, these variations lead to differential swelling, resulting in controlled deformation of the printed structures. This approach enables the programming of deformation pathways during fabrication, providing a simple and versatile framework for in situ shape-morphing.

In this proof-of-concept study, the induced differences in crosslinking density were sufficient to produce controlled bending of the constructs. Although demonstrated on simplified geometries, the strategy establishes a basis for more spatially resolved and potentially volumetric shape-morphing architectures in hydrogel-based bioprinting.

## 2. Results and Discussion

### 2.1. GelMA Characterization

**Figure 1:**
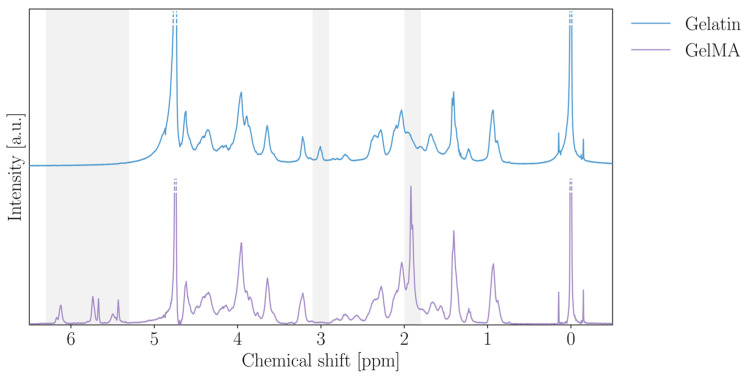
^1^H-NMR spectra of gelatin and GelMA.

Figure 1 shows the ^1^H-NMR spectra of gelatin and GelMA, obtained according to a previously reported protocol [40]. The two prominent peaks at 0 ppm and 4.79 ppm correspond to the TMSP internal standard and the solvent peak (D_2_O), respectively. In the GelMA spectrum, characteristic signals are observed in the regions of 6.50–5.00 ppm and 2.00–1.85 ppm, corresponding to the acrylic and methyl protons of the methacrylic groups introduced during the methacrylation reaction. These peaks are absent in native gelatin, confirming that methacryloyl groups have been successfully grafted onto the gelatin backbone.

Additional confirmation of functionalization is provided by the disappearance of the peak at 2.80 ppm, assigned to the methylene protons of free lysine residues. This signal is clearly visible in the gelatin spectrum but absent in GelMA, indicating that the lysine amine groups have been fully consumed during the reaction.

Moreover, peaks at 6.16 and 5.77 ppm are attributed to acrylic protons of methacrylate groups, while peaks at 5.72 and 5.47 ppm correspond to methacrylamide groups. These signals indicate that methacrylic anhydride reacted not only with lysine residues but also with hydroxyl groups. This broad reactivity reflects the high amount of methacrylic anhydride used during synthesis, which promotes extensive functionalization at multiple sites along the gelatin chain.

The degree of methacrylation (DM) of the synthesized GelMA was quantified as 0.36 ± 0.02 mmol/g, corresponding to a high degree of functionalization [41]. The measurement was performed across three independent batches, showing limited variability (standard deviation of approximately 5%) and confirming the reproducibility of the synthesis. Such a DM value is particularly advantageous for the intended application. A higher concentration of methacryloyl groups increases the density of polymerizable sites, enabling the formation of a mechanically stable network even at lower overall degrees of crosslinking [42]. This extends the range of effective exposure times during printing, providing greater flexibility in tuning the material’s mechanical properties and swelling behavior. Such tunability is essential for shape-morphing applications, where controlled differential swelling is required.

Additionally, a higher density of reactive groups improves the polymerization kinetics, allowing for shorter UV exposure times to achieve the same network density. This results in faster printing speeds and reduced phototoxicity for encapsulated cells, thereby enhancing biocompatibility [43].

Finally, a high degree of methacrylation enhances printing resolution. The dense distribution of crosslinkable sites ensures that free radicals generated by the photoinitiator are rapidly consumed in the immediate vicinity, limiting their diffusion and reducing unintended crosslinking in surrounding areas. This effect is especially important at longer exposure times: highly substituted hydrogels retain local reactivity, whereas poorly substituted ones allow radical migration, leading to a loss of spatial fidelity [44].

### 2.2. Printability Assessment

The designed geometry was successfully printed at exposure times ranging from 30 to 70 s. At 20 and 25 s, the results were inconsistent—some prints were completed successfully, while others exhibited missing features.

**Figure 2:**
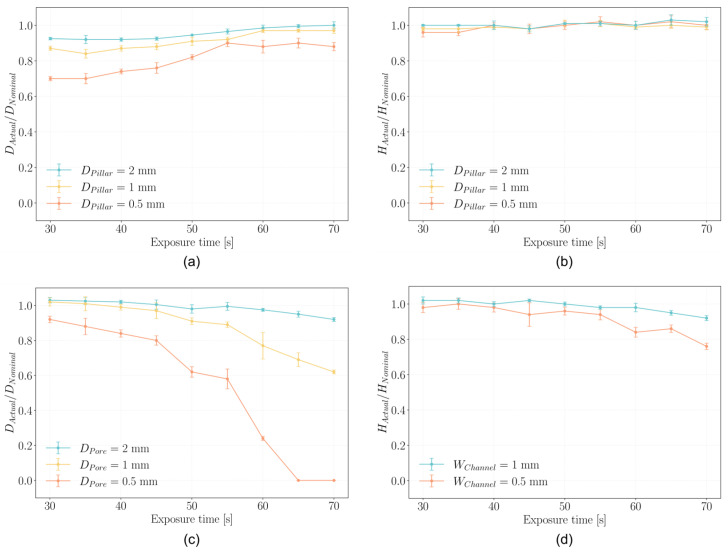
Printability assessment varying exposure time per layer. (**a**) Accuracy of pillars diameter; (**b**) Pillars integrity; (**c**) Accuracy of pores diameter; (**d**) Accuracy of horizontal channels height.

The accuracy of the printed pillar diameters (Figure 2a) was found to be below unity at shorter exposure times, indicating undersized structures relative to the designed dimensions. Accuracy approached unity as exposure time increased, with the effect being particularly pronounced for the smallest features, such as the 0.5 mm pillars (Figure 2a). This behavior is likely attributable to an insufficient degree of crosslinking at lower exposure times, which fails to preserve the sharp edges and fine details of smaller geometries [45].

The inclusion of tartrazine further contributes to this effect by absorbing part of the UV radiation, delaying the initiation of photocuring and thus slowing down the polymerization rate. Nevertheless, accuracy remained above 70% across all exposure times, indicating a consistent and reasonably good agreement between the printed and designed geometries. In DLP-based bioprinting, deviations from the nominal design are inherently expected due to photopolymerization phenomena such as light scattering and overcuring [46,47]. Within this context, the observed level of accuracy can be considered satisfactory, as moderate discrepancies can be systematically mitigated through design compensation strategies (e.g., CAD-based corrections). Conversely, larger deviations would compromise geometric fidelity to an extent that cannot be effectively corrected, ultimately limiting the reliability of the fabrication process.

Notably, even at higher exposure times, accuracy did not exceed 100%, suggesting that no unintended crosslinking occurred beyond the intended geometry (i.e., pillars did not become oversized). This outcome indicates that the combination of tartrazine and a high degree of GelMA methacrylation effectively confines crosslinking within the xy plane, preserving the fidelity of printed features.

In contrast to lateral dimensions, pillar height (Figure 2b) demonstrated excellent fidelity, with the ratio between the actual and designed values consistently close to unity across all tested exposure times. This indicates that, within the selected range, variations in exposure time do not significantly affect vertical accuracy. This stability can be attributed to the fact that resolution along the z-direction is primarily dictated by the predefined layer thickness, rather than by exposure time or material-specific optimization. As long as the degree of polymerization is sufficient to solidify each layer, further exposure does not impact the final height.

Unlike lateral dimensions, which are sensitive to light scattering, diffusion of free radicals, and over-polymerization, the z-resolution benefits from the inherently layered nature of the printing process, which compartmentalizes each vertical increment and shields it from cumulative effects [10].

Moreover, the fact that all pillars were successfully printed to their full height across all exposure times supports the robustness and repeatability of the process. It suggests reliable layer-by-layer curing and efficient light penetration throughout the construct, even at lower exposure times.

An opposite trend is observed for hole diameters (Figure 2c), where the ratio between actual and nominal values decreases with increasing exposure time, indicating unwanted lateral polymerization. This effect is minimal at shorter exposure times, with accuracy remaining above 80% up to 45 s for all features. However, it becomes more pronounced at higher exposure times, particularly for smaller holes.

This behavior is characteristic of vat photopolymerization, which is inherently influenced by light scattering within the bioink. Scattered light can deviate from the intended path and reach unexposed regions—such as internal voids or channels—causing out-of-target polymerization. As a result, holes and channels may partially close, compromising resolution and geometric fidelity.

Although the addition of tartrazine contributes to reducing light scattering by absorbing excess radiation, it cannot fully prevent this phenomenon during prolonged exposures [48].

A slight deviation from the monotonic decrease is observed between 50 and 55 s for all pore sizes. While this variation remains within the experimental uncertainty, the overall trend suggests the presence of two distinct regimes. Up to 50 s, the decrease in pore size is relatively gradual, which may be associated with overcuring driven by radical diffusion from directly irradiated regions. Beyond 55 s, a more pronounced reduction in accuracy is observed, particularly for smaller pores, where complete closure occurs. This sharper decrease may indicate the onset of an additional overcuring mechanism, likely related to secondary light exposure (e.g., scattering), which can activate polymerization in surrounding regions over longer exposure times.

In the intermediate range (50–55 s), a temporary stabilization of the pore size is observed, suggesting a transition between these two regimes. In this region, the contribution of diffusive overcuring may approach saturation, while secondary light-induced effects are not yet fully dominant, resulting in a balance between competing mechanisms. Although this interpretation remains qualitative, it is consistent with the observed change in slope across the investigated exposure range. Overall, the expected reduction in pore size with increasing exposure time is clearly preserved.

The internal height of the printed horizontal channels (Figure 2d) closely matches the nominal value across all tested exposure times, including in the case of the smallest channel height of 0.5 mm. This consistent vertical accuracy indicates that the curing of successive layers does not cause excessive or unintended curing in layers that have already been printed and solidified. Such behavior indicates the absence of significant z-overcuring and suggests that crosslinking remains well confined within the intended layer boundaries. The fact that open horizontal channels remain unobstructed, without closure caused by curing of the overlying layers, indicates effective confinement of light in the vertical (z) direction.

Indeed, during the photopolymerization of successive layers, light penetration into previously printed regions may lead to unintended additional crosslinking. This effect could compromise vertical resolution and, more critically, undermine the ability to control the local degree of crosslinking, an essential feature enabling the shape-morphing strategy adopted in this work.

Interestingly, this behavior contrasts with that observed for pores (Figure 2c), which show a progressive reduction in diameter with increasing exposure time. This difference can be attributed to the distinct degree of geometric confinement: pores are confined in two directions and may retain the same precursor volume over successive layers, promoting cumulative unintended polymerization due to scattered light, whereas channels are less confined and more effectively refreshed by the surrounding resin, limiting this effect.

Moreover, the fact that the channels are consistently closed at the top without any loss of structural integrity indicates that overhanging features up to 1 mm in span can be reliably fabricated. This is especially notable considering the inherent challenges of printing unsupported geometries using LCD-based photopolymerization and hydrogel-based materials. Achieving this level of fidelity demonstrates a robust printing behavior under the tested conditions.

Overall, the printed structures exhibit good resolution across most of the investigated features and exposure conditions. However, at higher exposure times, the smallest pores show a progressive loss of accuracy, eventually leading to partial or complete closure due to overcuring effects. Nevertheless, achieving this level of precision remains noteworthy given the use of LCD-based photopolymerization with hydrogels, a combination often associated with challenges in accurately reproducing fine geometries [10]. Importantly, the observed resolution is not limited to a narrow set of processing conditions but is maintained over a broad range of exposure times. This indicates a robust and versatile printing process, capable of balancing geometric fidelity with tunable parameters. Such flexibility is essential for imparting advanced functionalities to the printed constructs, including spatially programmed behaviors such as shape-morphing.

### 2.3. Swelling Characterization

**Figure 3:**
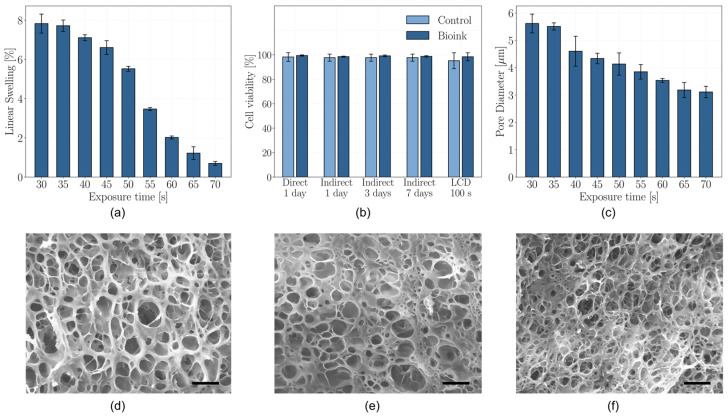
(**a**) Linear swelling as a function of exposure time per layer; (**b**) Cell viability obtained from different cytotoxicity assays; (**c**)Average pore diameter as a function of exposure time per layer; SEM image of microstructure obtained with (**d**) 30 s, (**e**) 50 s and (**f**) 70 s exposure per layer. Scale bars: 20 µm.

The linear expansion of the printed samples after 24 h of immersion in culture medium is reported in Figure 3a and Table 1 for each exposure time tested.

The swollen condition after 24 h was considered the most informative, as it reflects both the equilibrium configuration relevant for shape-morphing and the conditions experienced by cells in a biological environment [49].

The results reveal a clear trend: the linear swelling ratio of the hydrogel samples decreases with increasing exposure time per layer during the printing process. This behavior highlights the critical role of exposure time in modulating the swelling properties of the hydrogel, primarily through its effect on the polymer network density.

Specifically, longer exposure times result in a higher degree of photopolymerization, leading to the formation of a denser and more tightly crosslinked polymer network. This increased crosslinking density restricts the mobility of polymer chains and reduces the available free volume within the hydrogel matrix, thereby limiting water uptake and expansion. As a consequence, samples printed with shorter exposure times exhibit more pronounced swelling, while those fabricated with longer exposure times demonstrate a more constrained expansion behavior [50].

These findings are particularly relevant in the context of this study, which explores the potential for shape-morphing via differential swelling. The range of swelling ratios observed—while not excessive to compromise structural integrity or dimensional accuracy—is sufficiently broad to serve as an effective driving mechanism for shape transformation. Notably, the sample printed with a 30 s exposure exhibited the highest swelling ratio at 7.7%, whereas the sample printed with a 70 s exposure showed only 1% linear expansion, corresponding to the lowest swelling coefficient.

Importantly, all samples printed uniformly with a constant exposure time remained flat after immersion, with no observable bending. This indicates that photopolymerization was effectively confined to the intended regions during printing. In contrast, if uncontrolled light penetration had occurred—causing unintended crosslinking in previously cured layers—a gradient in crosslinking density would have developed across the sample thickness, leading to differential swelling and subsequent bending. The absence of such effects further supports the spatial control over crosslinking achieved in this process, both laterally and in the z-direction, which is essential for the reproducible fabrication of shape-morphing hydrogel constructs.

As regards the gel fraction, an increasing trend with exposure time is observed, with values rising from approximately 70% to 80%, as reported in Table 1. This behavior indicates a progressive increase in the fraction of polymer effectively incorporated into the crosslinked network, with a corresponding reduction in the soluble fraction. The observed trend is consistent with the expected effect of prolonged UV exposure, which enhances radical generation and promotes more extensive network formation.

Although the variation is relatively moderate, it reflects a measurable increase in network integrity within the investigated exposure window, suggesting that the crosslinking process is not fully saturated at shorter exposure times. This incomplete saturation suggests sufficient tunability in the system to introduce spatial variations in crosslinking, which is a key requirement for achieving differential swelling and the resulting shape-morphing behavior.

### 2.4. Mechanical Properties and Crosslinking Density

**Figure 4:**
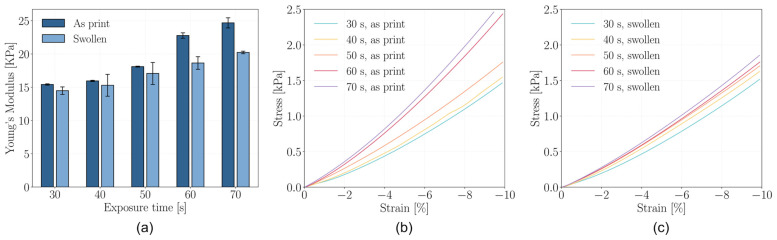
(**a**) Young’s modulus as a function of exposure time per layer of pristine and swollen samples; Stress–strain curves of compression test on (**b**) pristine and (**c**) swollen samples.

The elastic moduli of the hydrogels, as a function of the UV exposure time employed during printing, are presented in Figure 4a, both in the as-printed state and after 24 h of immersion in culture medium. Compression testing reveals a clear trend of increasing stiffness with longer exposure times, attributable to the higher degree of crosslinking achieved through prolonged UV exposure. This results in a more densely interconnected polymer network, which enhances the stiffness of the hydrogel [51].

Following immersion in culture medium, all samples exhibit a reduction in elastic modulus due to hydrogel swelling. Water uptake increases the network’s free volume, reducing the polymer volume fraction and expanding the distance between crosslinks, thereby diminishing mechanical strength [52]. This effect is consistent across all exposure conditions, confirming the hydrogel’s capacity to equilibrate with its environment while maintaining structural cohesion.

Importantly, the obtained modulus values, in the order of ~20 kPa for GelMA-based hydrogels, are consistent with reports from the literature [53,54] and fall within the desirable range for several biomedical applications. In particular, such stiffness is compatible with the mechanical microenvironment required for muscle and connective tissue engineering [55], and can also support early-stage bone regeneration [56].

A noteworthy result is the relatively limited variation in elastic modulus across the range of exposure times investigated. Although crosslinking density is clearly influenced by UV dose, and such differences are essential to enable swelling-driven shape-morphing, the resulting mechanical variations remain within a narrow range.

This balance is particularly advantageous in the present approach. On one hand, sufficient differences in crosslinking density are preserved to induce controlled deformation; on the other, the absence of excessive mechanical heterogeneity ensures that the printed constructs remain mechanically coherent. This aspect is especially relevant for biomedical applications, as it is necessary for homogeneous cell proliferation and reduces the risk of localized stress-induced alterations in cell behavior.

Beyond the general decrease in elastic modulus following immersion in culture medium, all hydrogels maintained a mechanical response similar to the pristine state also after 24 h of swelling. Although water absorption induces softening by increasing the polymer network’s free volume and decreasing the crosslink density per unit volume, the hydrogels maintained elastic moduli within a functional range. This is particularly relevant in the context of biofabrication, where printed constructs are expected to remain mechanically stable in hydrated conditions resembling in vivo environments.

The compressive stress–strain curves (Figure 4b,c), which were recorded up to 10% strain (the limit imposed by the experimental setup rather than by material failure), show continuous and monotonic stress development without evidence of fracture or collapse. This observation holds for all exposure times, including those associated with the highest degrees of crosslinking. The absence of abrupt transitions in the stress response suggests that increased stiffness due to longer exposure does not lead to embrittlement, which is a common concern when optimizing crosslink density in hydrogel-based systems.

This mechanical behavior indicates that the printed hydrogels are capable of sustaining moderate mechanical loads without undergoing failure, even after swelling. Such performance ensures that structural integrity is preserved during post-printing handling, swelling-driven actuation, or potential mechanical stimulation in biological applications. Furthermore, this combination of stiffness tunability and retained mechanical behaviour offers important advantages for applications in tissue engineering, particularly in contexts where both shape-morphing and mechanical integrity are required. The ability to locally modulate crosslinking without introducing weak or failure-prone regions is essential for fabricating constructs with spatially programmed responses or region-specific functions.

The effective crosslinking density of the hydrogels was estimated from the elastic modulus in the swollen state using rubber elasticity theory, and the results are reported in Table 1. It should be noted that this parameter does not represent the absolute number of chemical crosslinks, but rather the density of elastically active network chains contributing to the mechanical response of the hydrogel.

Within this framework, the estimated crosslinking density increases with increasing exposure time. This trend is consistent with the expected higher degree of photocrosslinking at longer exposure times, which promotes the formation of a more tightly connected polymer network. The results therefore confirm that the applied exposure conditions effectively modulate the network structure, leading to progressively higher crosslinking densities.

### 2.5. Microstructure Characterization

SEM imaging of freeze-dried GelMA hydrogel samples after 24 h of swelling revealed a clear correlation between microstructural morphology and the exposure time applied during the printing process. Specifically, samples printed with an exposure time of 30 s exhibited an average pore diameter of 5.62 ± 0.34 µm, while those fabricated with 70 s exposure showed significantly smaller pores, averaging 3.11 ± 0.21 µm (Figure 3b,d–f). Shorter exposure times resulted in a lower pore density and larger individual pores, whereas longer exposure durations led to a denser porous network with reduced pore size.

These observations reinforce the structural trends identified in both swelling and mechanical characterizations, confirming the role of exposure time in modulating the hydrogel network architecture. Increased exposure duration enhances the degree of crosslinking, thereby producing a more tightly interconnected polymer network, which manifests as a finer pore structure [57]. The spatial arrangement of pores is known to influence both mechanical response and swelling capability. A denser microstructure with smaller pores generally corresponds to a stiffer material due to higher crosslinking density [58], while simultaneously restricting the ingress of water, thus reducing swelling [59].

The aforementioned relationships are supported by the experimental findings: samples with larger pores, resulting from shorter exposure times, demonstrated higher swelling ratios and lower compressive modulus, while samples with smaller pores showed reduced swelling and enhanced mechanical stiffness. This underscores the direct impact of microarchitecture on functional performance.

Beyond mechanical and swelling behavior, scaffold porosity plays a critical role in regulating cell adhesion, proliferation, and overall tissue integration [53]. Smaller pore sizes, at an approximately constant solid fraction (and thus similar porosity), lead to a larger total internal surface area for a given volume, as a greater number of smaller pores is required to occupy the same void space. This increased surface area can enhance initial cell attachment, but may also limit proliferation due to early contact inhibition. Conversely, highly porous and interconnected networks facilitate nutrient and oxygen diffusion, enhance waste transport, and enable the controlled release of bioactive agents. However, excessive pore enlargement can compromise mechanical integrity, potentially leading to mismatches with native tissue properties [60].

Therefore, careful tuning of scaffold porosity is essential to achieve an optimal balance between biological performance and structural stability. The ideal pore size, evaluated by imaging of freeze-dried constructs, is strongly tissue-dependent and may vary depending on the system, with ranges reported in the literature including approximately 5 µm for neovascularization, 5–15 µm for fibroblast ingrowth, 20–125 µm for skin regeneration, 40–100 µm for osteoid formation, 100–350 µm for bone regeneration, and around 20 µm for hepatocyte infiltration [61].

In this study, the measured pore sizes fall within the range appropriate for fibroblast culture. While larger pores may be preferable to promote deeper infiltration and proliferation, the pore size distribution observed, characterized by both dominant larger pores and a population of smaller ones, ensures sufficient permeability for cell growth and nutrient diffusion. Such findings are consistent with the potential use of the printed GelMA hydrogels for applications involving soft tissue regeneration.

### 2.6. Cell Viability

Direct cytotoxicity assay, indirect cytotoxicity assay and UV exposure of cell-laden hydrogel were performed to assess the potential cytotoxicity of the bioink and the printing process. In all conditions tested, cell viability exceeded 95%, with no statistically significant differences observed between formulations or processing conditions, as *p*-values consistently remained above 0.05 (Figure 3b). Therefore, the synthesized GelMA hydrogel is free of residual reaction byproducts, which would otherwise likely induce cytotoxic effects even after brief exposure. Furthermore, the bioink itself was demonstrated to be non-cytotoxic, reinforcing its suitability for cell-laden bioprinting applications.

Importantly, UV exposure associated with LCD-based photopolymerization did not adversely affect cell viability under the tested conditions, suggesting that both the material composition and the applied processing parameters are compatible with maintaining cell health during bioprinting. This finding is notable given the known potential for UV radiation to cause cellular damage during photopolymerization processes. Additionally, the presence of tartrazine at concentrations higher than typically reported in the literature did not contribute to cytotoxicity; rather, it may provide a protective effect by absorbing UV radiation, thereby mitigating potential phototoxic effects on encapsulated cells.

At the end of direct cytotoxicity assay, after the 24 h incubation period, cells retained their characteristic spindle-shaped morphology both directly beneath the hydrogel disk and in the surrounding culture medium, indicating that direct contact with the material did not alter cellular behavior or induce morphological changes such as impaired spreading, elongation, or attachment. Moreover, no signs of cellular stress—including rounding, shrinkage, or detachment—were observed, further supporting the conclusion that the material is biocompatible and does not interfere with normal cell adhesion.

The even distribution of viable cells around and underneath the hydrogel further suggests the absence of soluble cytotoxic factors released by the material. Upon removal of the hydrogel disks, a substantial number of adherent fibroblasts remained attached to the hydrogel surface, highlighting a strong affinity between the bioink and cells. This property is essential for bioprinting applications, where maintaining robust cell-material interactions is critical to ensuring the structural integrity and functional stability of printed constructs.

The fabrication of cell-laden printed constructs was not explored within the present study, as previous literature demonstrated both LCD-based bioprinting of GelMA [62] and the feasibility of cell-laden shape-morphing hydrogel systems [63]. Accordingly, the focus was placed on validating the proposed strategy at the material and process level.

### 2.7. Shape-Morphing

**Figure 5:**
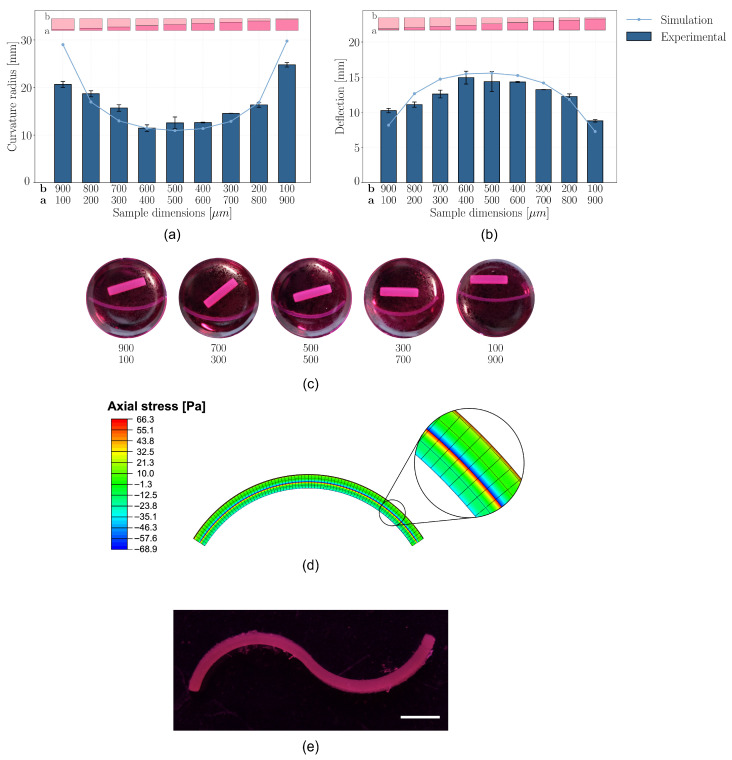
(**a**) Radius of curvature and (**b**) deflection of bilayer shape-morphing samples: comparison between experimental measurements and simulations; (**c**) Bilayer shape-morphing samples after 24 h in culture medium; (**d**) Simulation results showing predicted deformed configuration and stress state for the 500–500 sample; (**e**) S-shape sample, showing opposite curvature regions after 24 h in culture medium; scale bar: 5 mm.

The originally parallelepiped-shaped GelMA samples used for shape-morphing assessment exhibited a distinct bending behavior (Figure 5a–c) following immersion in culture medium for 24 h. These samples were printed by dividing their height into two portions: the lower portion (a) was polymerized with an exposure time of 65 s per layer, while the upper portion (b) received a shorter exposure of 35 s per layer.

The difference in exposure time resulted in a variation in crosslinking. The lower region, exposed for longer times, developed a higher crosslink density and a denser polymer network. Conversely, the upper portion, exposed for a shorter duration, was less crosslinked, yielding a looser network structure.

Upon immersion in an aqueous environment, the swelling behavior of the two regions diverged due to their respective crosslinking densities, as previously described. The upper, less crosslinked region contained more free volume within its polymer matrix, enabling it to absorb greater amounts of water and expand significantly. In contrast, the lower, more crosslinked region exhibited a denser network with fewer available free spaces for solvent penetration, resulting in reduced swelling. This differential swelling induced an internal mechanical imbalance within the bilayer hydrogel.

As the upper region swelled more extensively than the lower region, it experienced a compressive state of stress due to the adhesion to the relatively rigid and less swollen lower portion. Simultaneously, the lower region experienced tensile stress as it was stretched by the expansion of the upper layer. This distribution of stresses generated a bending moment, causing the sample to curve towards the more crosslinked, less swollen side. The magnitude of bending was quantified by measuring the curvature radius along the outer perimeter.

Samples with balanced height ratios (in which region a and b showed similar thickness) demonstrated a more pronounced bending response, as both regions contributed substantially to the overall internal state of stress, resulting in a stronger bending moment. The opposing compressive and tensile forces led to a higher degree of curvature as the sample reached mechanical equilibrium.

Conversely, samples with highly unbalanced height ratios exhibited diminished bending. When the more crosslinked lower region was substantially thicker, it dominated the mechanical response, thereby imposing the swelling-induced deformation of the upper layer to the whole construct. In contrast, if the less crosslinked upper region was disproportionately thick, the swelling-induced expansion was more broadly distributed, with only a limited cross-sectional area of the lower region available to resist stretching. In both scenarios, the imbalance in thickness diminished the differential swelling effect, ultimately hindering effective shape-morphing.

To qualitatively demonstrate the extension of the proposed strategy to more complex geometries, a proof-of-concept structure with spatially programmed crosslinking gradients was fabricated. The construct was designed to induce opposite swelling-induced bending in adjacent regions by inverting the exposure profile between the two halves. Upon immersion in DMEM, the sample exhibited the expected deformation, with each half bending in opposite directions (Figure 5e) as a result of the local differences in crosslinking degree. This differential response led to the formation of an overall S-shaped configuration, confirming the ability to encode complex, multi-directional shape transformations within a single construct. Overall, these results highlight the versatility of the approach and its potential for the rational design of more sophisticated morphing architectures, demonstrating how controlled spatial modulation of crosslinking can be effectively leveraged to achieve predictable and programmable shape changes.

Further investigations can be carried out to achieve lower radii of curvature, i.e., increase the magnitude of bending. One approach to enhance bending involves reducing the overall sample thickness while maintaining thickness balance between layers [34]. This reduced thickness decreases bending stiffness of the structure by lowering the moment of inertia, thereby enabling greater curvature.

Finally, chemical modification of GelMA to increase hydrophilicity or copolymerization/blending with more hydrophilic polymers represents another promising route. Such modifications should be designed to preserve mechanical properties—including stiffness and structural integrity—while enhancing water absorption and swelling-induced bending.

### 2.8. Shape-Morphing Simulation

The observed bending behavior was simulated by modeling the phenomenon as a thermal expansion problem. The two regions of the hydrogel were assigned mechanical properties and swelling capacities expressed through an equivalent thermal expansion coefficient, corresponding to their experimentally characterized properties.

The simulated samples exhibited bending with the concavity oriented toward the more crosslinked region (Figure 5d), in agreement with experimental observations. The highest stress was localized at the interface between the two regions, where the mismatch in expansion generated a concentration of mechanical stress. Analysis of the axial stress distribution revealed that the less crosslinked region was subjected to compressive stresses, while the more crosslinked region experienced tensile stresses, further confirming the internal stress gradient responsible for the curvature.

The radii of curvature predicted by the simulation closely matched the experimental measurements (Figure 5a). Minor discrepancies were observed for samples with highly unbalanced layer distributions (ratios of 900:100 and 100:900), which also corresponded to the least curved samples in both the experimental and simulated datasets. These small differences are attributed to experimental variability: radius measurements were performed using images of the samples floating freely in culture medium to preserve their equilibrium configuration. However, slight sample displacements during handling could introduce variations to some extent. In near-flat samples, even minimal deviations can result in large apparent differences in curvature radius due to the asymptotic nature of the metric—thus, the presence of experimental noise in such cases is expected.

By contrast, samples with more balanced height ratios—and consequently more pronounced curvature—showed a strong agreement between simulated and experimental radii of curvature.

When considering deflection instead of radius of curvature as the comparative metric (Figure 5b), the overall trend remains consistent, as do the observed discrepancies. However, deflection proves to be a more robust parameter, being less sensitive to minor variations in sample positioning and image acquisition. This is particularly relevant in the case of near-flat samples, where small geometric deviations can lead to disproportionately large changes in calculated curvature radius.

Quantitative analysis revealed that the simulated-to-experimental deflection ratio was at least 80% for the most unbalanced samples (900:100 and 100:900), while for all other samples, this ratio approached 100%, indicating excellent agreement.

These results confirm that the shape-morphing phenomenon induced by differential crosslinking is successfully captured by the simulation. The direction and magnitude of the bending, as well as the stress distribution, closely align with experimental observations, particularly in samples with balanced thickness ratios. This coherence validates the effectiveness of the overall strategy used to program shape-morphing behavior, including the careful selection of material and process parameters to locally control the degree of crosslinking, as well as the accuracy of the adopted simulation approach. Notably, the simulations were conducted under the assumption of a sharp interface between two regions with distinct mechanical and swelling properties. The strong agreement with experimental results is consistent with the expected crosslinking profile and suggests that the morphing occurred following the proposed mechanisms. The use of thermal expansion as an analog for swelling proved to be a robust and reliable modeling strategy. Furthermore, the close match in deflection between simulations and experiments corroborates the appropriateness of the assigned material properties and swelling coefficients.

The reported findings demonstrate not only the reliability of the computational framework but also the effectiveness of a novel shape-morphing strategy based on differential crosslinking through LCD-based 3D printing. The approach enables precise control over the final geometry through localized modulation of material properties and offers strong predictive power, both essential for advancing 4D bioprinting. The close agreement between simulations and experiments confirms that the intended morphing behavior was successfully programmed and reproduced, supporting the methodology. With this validated framework in place, future work can focus on enhancing the degree of shape transformation and on the design and characterization of application-specific structures for biomedical and clinical use, where programmable geometry is a key functional requirement.

## 3. Conclusions

This study presents a proof-of-concept 4D bioprinting strategy based on swelling-driven shape-morphing in hydrogels, achieved through layer-by-layer modulation of crosslinking density during vat photopolymerization. By varying exposure time across successive layers, controlled deformations were induced upon immersion in aqueous media, enabling in situ shape programming without the need for additional materials or external stimuli.

Compared to existing approaches, often limited to thin structures or externally imposed gradients, this method provides a simple and versatile framework based on a parameter intrinsic to the printing process. Finite element simulations and experimental characterization confirmed the underlying mechanism and demonstrated compatibility with high-resolution fabrication and cell-laden constructs.

While only simple geometries were explored in this work, the proposed strategy can be extended to more complex architectures through spatial control of exposure conditions. In addition, although the material formulation was not specifically optimized, the generality of the approach suggests potential applicability to other photocurable hydrogel systems. The fabrication of cell-laden constructs was not addressed within the present study and is considered an expected extension of this work. Given the demonstrated cytocompatibility of the material and processing conditions, along with prior reports on both LCD-based GelMA bioprinting and cell-laden shape-morphing hydrogels, the integration of living cells within this framework appears feasible. The inclusion of cell-laden constructs will be particularly relevant when applying this strategy to novel material systems or when targeting specific tissue engineering applications.

Overall, these findings establish a foundation for in situ programmable shape-morphing functionality in hydrogel-based constructs, highlighting the potential of exposure-controlled crosslinking as a flexible tool for 4D bioprinting and the development of adaptive biomedical systems.

## 4. Materials and Methods

The proposed method is based on locally varying the exposure time on a layer-by-layer basis during vat photopolymerization to induce shape-morphing in hydrogel constructs.

Within each printed layer, the exposure time can be varied across the plane, generating regions with different crosslinking densities. By repeating this process across successive layers, a three-dimensional distribution of crosslinking density is obtained. Upon immersion in aqueous media, such spatial variations induce differential swelling and result in controlled deformation of the constructs.

To identify a suitable exposure range, samples were first fabricated using a uniform exposure time, varied between 20 and 70 s, and their shape fidelity was evaluated. Since the morphing behavior depends on both swelling and mechanical response, linear swelling and mechanical properties were characterized for each condition. In addition, microstructure (porosity) and cytocompatibility were assessed to evaluate suitability for biomedical applications.

Proof-of-concept morphing experiments were then performed by introducing a vertical gradient in exposure time, with a more crosslinked bottom region and a less crosslinked top region. Finally, finite element simulations incorporating the experimentally measured swelling and mechanical properties were conducted to predict and rationalize the observed deformations.

### 4.1. Materials

Type A gelatin from porcine skin (high gel strength, Millipore, Burlington, MA, USA), Carbonate-Bicarbonate.

(CB) buffer, methacrylic anhydride (MA), phosphate-buffered saline (PBS, pH 7.4), cellulose dialysis tubing (cut-off molecular weight = 14 kDa), lithium phenyl-2,4,6-trimethylbenzoylphosphinate (LAP), tartrazine (dye content 85%), Dulbecco’s Modified Eagle’s Medium (DMEM, high glucose, with sodium bicarbonate, sodium pyruvate, without glutamine), deuterated water containing trimethylsilylpropionic acid standard (0.75% *w*/*w*), Trypan blue (solution, 0.4%) were purchased from Sigma-Aldrich (Saint Louis, MI, USA).

### 4.2. GelMA Synthesis and Characterization

Gelatin methacryloyl (GelMA) was synthesized similarly to established methods from literature [64]. A 10% *w*/*v* gelatin solution was prepared in CB buffer (pH = 9.0, 0.25 M) at 50 °C under constant stirring. MA (3% *v*/*v*) was added dropwise, the pH was adjusted to 9 using 10 M NaOH and the reaction proceeded for 3 h under stirring, adjusting the pH to 9 every 30 min. The reaction was then quenched by adjusting the pH to 7.4 using 5 M HCl. Purification was performed via dialysis (37 °C, 3 days, daily water changes) against distilled water. The dialyzed solution was filtered to remove possible particulates, freeze-dried, and stored at −18 °C until use.

The quantification of the degree of substitution of the synthesized GelMA was performed using a well-known technique described in the literature [65], exploiting spectra obtained from proton nuclear magnetic resonance.

Sample preparation for the H-NMR analysis followed this procedure: 15 mg of gelatin and 15 mg of GelMA were individually dissolved in 600 μL of deuterated water containing the trimethylsilylpropionic acid standard (0.75% *w*/*w*). ^1^H NMR (400 MHz) spectra were recorded on a Bruker Avance 400 (Bruker, Billerica, MA, USA) and were processed using the MestreNova v14.1.0 software.

The degree of methacrylation (DM) shows the amount of methacrylic groups grafted onto gelatin chains during the reaction [66]. DM was calculated, using spectra normalized with TMSP as the internal standard [67], with the 0 ppm peak set to 1, as:

(1) $$DM_{TMSP}=\frac{\int Meth}{\int TMSP}\cdot \frac{9H}{1H}\cdot \frac{n_{TMSP}\;\left[mmol\right]}{m_{GelMA}\;[g]}$$

in which H denotes hydrogens, $n_{TMSP}$ represents the moles of TMSP, and $m_{GelMA}$ is the mass of GelMA. The methacrylic group integration area is defined by the peaks between 5.80 and 5.30 ppm, while the TMSP peak at 0 ppm integrates over 9 protons. The number of moles of TMSP is based on the volume of D_2_O used (600 μL), and the mass of GelMA is known.

### 4.3. Hydrogel Formulation

The hydrogel precursor solution was prepared by dissolving GelMA (15% *w*/*v*), LAP (1% *w*/*v*) as photoinitiator, and tartrazine (1.5 mM) as a UV absorber in PBS (pH 7.4). The formulation was designed to enable precise, layer-by-layer control of the photocrosslinking process, with the aim of achieving spatially heterogeneous crosslinking densities. This gradient in crosslinking allows for region-specific swelling behavior, which is essential for inducing programmed shape-morphing. To enhance confinement of UV light and improve resolution across layers, a higher concentration of tartrazine was used compared to typical literature values [10]. Additionally, relatively high [10] LAP content was selected to ensure effective crosslinking within practical exposure times, despite the increased light attenuation introduced by the absorber.

The selection of these concentrations was guided by a DoE-based optical analysis, including the estimation of an effective attenuation coefficient and its projection at the printing scale; full details are provided in the Supplementary Information.

In this work, a conventional hydrogel formulation was employed, and the focus was placed on demonstrating the feasibility of the proposed strategy rather than optimizing the material formulation.

### 4.4. Printability Assessment

Printability was evaluated using a Frozen Sonic Mini 8K LCD printer (405 nm, 1.4 mW/cm^2^ light intensity, measured using a SpectriLight ILT950 spectroradiometer, International Light Technologies Inc., Peabody, MA, USA) equipped with a reduced-volume vat and a temperature and humidity control system. Printing was performed at 30 °C to maintain the hydrogel in the liquid state and 90% relative humidity to minimize dehydration. Slicing was carried out using Chitubox PRO (CBD Technology Co., Ltd., Shenzhen, China).

To achieve spatially controlled crosslinking, the curing dose was modulated across different regions of the same print. The slicing software allowed for pixel-wise variation in exposure power, enabling selective tuning of the crosslinking degree within a single layer. For simplicity, equivalent exposure times (defined as power-percentage multiplied by exposure time) are reported. Printing parameters are listed in Table 2.

The “+” symbol used in some parameters indicates a two-step motion profile. For both lifting and retraction, the first value refers to the initial stage of the movement (near the vat for lifting and far from the vat for retraction), while the second value corresponds to the subsequent stage (far from the vat for lifting and near the vat for retraction).

The exposure time range (20–70 s) was selected based on preliminary trials. Below 20 s, the hydrogel did not achieve sufficient crosslinking to maintain structural integrity, while exposure times above 70 s led to poor printing reproducibility due to excessive adhesion to the vat, resulting in damage during the detachment phase.

**Figure 6:**
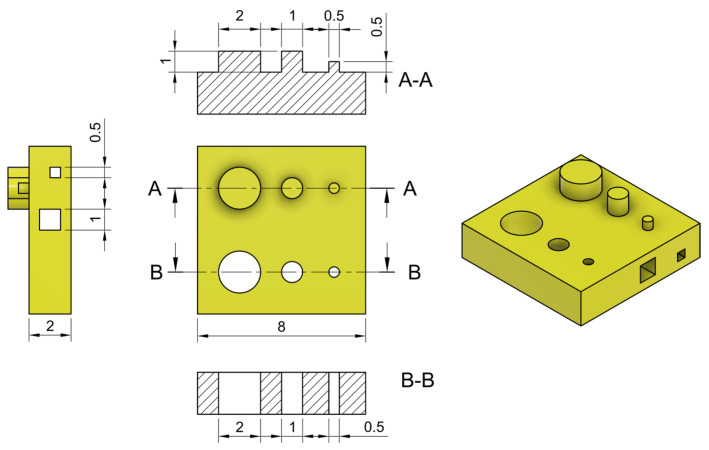
Trial geometry used for printability assessment.

To assess the exposure window and printing fidelity, a test geometry (Figure 6) was fabricated at varying exposure times, and critical dimensions were quantified using ImageJ v1.53a.

For vertical pillars, accuracy was defined as the ratio of actual to designed diameter, and integrity as the ratio of actual to designed height. For vertical holes and horizontal internal channels, accuracy was defined as the ratio of actual to designed diameter and height, respectively.

### 4.5. Swelling and Gel Fraction Characterization

To control shape-morphing via differential swelling within a single construct, the material’s swelling behavior was characterized as a function of crosslinking degree after immersion in DMEM. Parallelepiped samples (2 mm × 2 mm × 20 mm) were printed at varying exposure times (30 to 70 s) to modulate crosslinking density. Samples were then immersed in 3 mL of DMEM and incubated at 37 °C for 24 h. After incubation, the principal length of each sample was measured using ImageJ, and the swelling ratio was calculated as the relative increase in length along the main axis.

Following the swelling test, samples were dried in a vacuum oven at 60 °C until constant weight. The gel fraction was then determined as the ratio between the final dry mass and the theoretical polymer mass initially present in the sample.

### 4.6. Mechanical Properties and Crosslinking Density

Unconfined compression tests were conducted on printed hydrogels using a Discovery DHR2 stress-controlled rheometer (TA Instruments, New Castle, DE, USA) with a 20 mm stainless steel plate and Peltier temperature control, set at 37 °C. Cylindrical samples (10 mm diameter, 1.5 mm thickness) were printed at exposure times from 30 to 70 s. Tests were performed immediately after printing and after 24 h incubation in 3 mL DMEM at 37 °C. Actual sample diameters were measured prior testing via ImageJ to account for possible swelling or shrinkage. The initial test gap was set manually to ensure contact without any initial compression. Compression was applied at 100 µm/s using a 50 N load cell. The elastic modulus was calculated from the slope of the initial linear region of the stress–strain curve. The initial sample thickness was estimated as the x-intercept of the fitted linear portion to ensure consistency, considering the manually adjusted gap at test start.

The effective crosslinking density of the hydrogels in the swollen state was estimated from the elastic modulus using rubber elasticity theory, including phantom network correction and swelling effects. The relationship can be expressed as:

(2) $$G=\nu_{e}RT\left(1-\frac{2}{f}\right)\phi_{0}^{2/3}\phi_{s}^{1/3}$$

where G is the shear modulus in the swollen state, $\nu_{e}$ is the effective crosslinking density, R is the universal gas constant, T is the absolute temperature, f is the functionality of the crosslinks (assumed f=4 for GelMA hydrogels), $\phi_{0}$ is the polymer volume fraction in the reference (as prepared) state, and $\phi_{s}$ is the polymer volume fraction in the swollen state. The shear modulus was obtained from the measured compressive modulus assuming incompressibility (G=E/3) [68,69].

Volume fractions were evaluated as follows:

(3) $$\phi_{0}=\frac{\frac{w_{GelMA}}{\rho_{GelMA}}}{\frac{w_{GelMA}}{\rho_{GelMA}}+\frac{1-w_{GelMA}}{\rho_{water}}}$$

(4) $$\phi_{s}=\phi_{0}\cdot \frac{Gel\%}{{\left(1+LS\right)}^{3}}$$

where $w_{GelMA}$ is the initial GelMA weight fraction (15% *w*/*v*, therefore 13% *w*/*w*_Tot_), $\rho_{GelMA}$ is the density of GelMA (equal to 1.3 g cm^−3^ [70,71]), $\rho_{water}$ is the density of water, Gel% and LS are gel fraction and linear swelling evaluated in previous sections, respectively.

### 4.7. Microstructure Characterization

The microstructure of the printed GelMA hydrogels swelling was analyzed through scanning electron microscopy (Zeiss EVO 50EP extended-pressure SEM, Carl Zeiss S.p.A., Milano, Italy). Parallelepiped samples (2 mm × 2 mm × 10 mm) were printed with layer exposure times from 30 to 70 s and incubated in DMEM at 37 °C for 24 h. After swelling, samples were rapidly frozen in liquid nitrogen and fractured to expose the internal cross-section, followed by freeze-drying for 24 h.

Representative SEM images were acquired at 2000× magnification. Pore sizes within the GelMA microstructures were quantitatively assessed using ImageJ software. For each pore, two orthogonal diameters were manually measured, and their average value was used to represent the pore size. This procedure was repeated for all the pores visible within the analyzed area (approximately 40 × 40 µm), and the reported pore size corresponds to the average value calculated over all measured pores.

### 4.8. Cell Viability Assessment

Cytocompatibility of the bioink and the effects of 3D printing were evaluated using L929 mouse fibroblasts cultured under standard conditions (37 °C, 5% CO_2_, 95% humidity) in complete DMEM supplemented with 10% FBS, 1% Penicillin-Streptomycin, and 1% L-glutamine, with media refreshed every two days. Controls consisted of cells cultured in standard medium without exposure to bioink nor printing process. Experiments were performed in triplicate.

Direct cytotoxicity was assessed to evaluate immediate cytotoxicity of the bioink by seeding 10^5^ cells/well in 12-well plates and incubating to confluence, then placing crosslinked hydrogel disks (8 mm diameter) on the monolayer for 24 h. Post-incubation, cells were detached, mixed 1:1 with 0.4% Trypan Blue, and viability quantified via microscopy using a Neubauer chamber [72].

To examine potential longer-term cytotoxic effects, an indirect assay was performed. Crosslinked hydrogels were incubated in complete medium at 37 °C and 5% CO_2_, and conditioned medium was collected after 1, 3, and 7 days of incubation. Confluent L929 cultures were then exposed to conditioned medium or fresh medium (as a control) for 24 h before viability assessment as above [73].

To evaluate viability post-LCD vat photopolymerization, cell-laden bioink was placed in a 100 µm-depth Neubauer chamber, covered with a glass slip, and exposed upside-down to UV for 100 s. Viability was determined by Trypan Blue staining following exposure.

Statistical significance was determined by two-way ANOVA with *p* < 0.05.

### 4.9. Shape-Morphing

**Figure 7:**
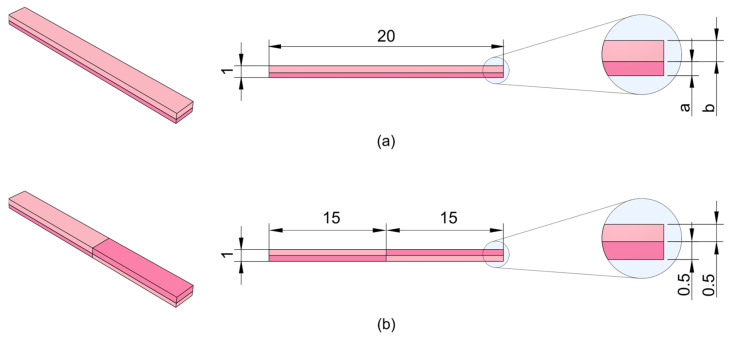
(**a**) Bilayer sample produced to assess shape-morphing behaviour; (**b**) Sample showing a double crosslinking gradient, designed to obtain a S-shaped final configuration upon swelling.

To evaluate shape-morphing behavior, parallelepiped-shaped samples were printed with two different exposure times within a single construct and incubated in culture medium at 37 °C for 24 h to monitor shape changes. Each sample measured 20 mm in length, 2 mm in width, and 1 mm in total height, divided into two regions with varying thicknesses (Figure 7a). The lower region, printed at 65 s exposure, varied in thickness from 100 µm to 900 µm in 100 µm increments, while the upper region, complementary in thickness (900 µm to 100 µm), was printed at 35 s exposure time.

After immersion in 3 mL of DMEM, samples were imaged and analyzed using ImageJ. Three coordinate points along the outer perimeter were recorded to quantify the curvature developed through geometrical analysis.

Finally, to provide a proof of concept of the extension of this strategy to more complex geometries, an additional sample was fabricated with spatially programmed crosslinking gradients. Specifically, one half of the construct was printed with a higher exposure time (65 s) in the bottom region and a lower exposure time (35 s) in the top region, while the other half was fabricated with the opposite configuration, i.e., higher exposure at the top and lower at the bottom (Figure 7b).

This arrangement was designed to induce opposite bending directions in the two halves upon swelling, leading to the formation of an overall S-shaped structure.

### 4.10. Shape-Morphing Simulation

Shape-morphing behavior was simulated using the Finite Element Method (FEM) in Abaqus 2024. The bilayer printed constructs, composed of two regions corresponding to different exposure times, were modeled as a bilayer structure made of two distinct materials. Each region was assigned a Young’s modulus derived from the compression tests: the region printed with 35 s exposure was modeled using the average modulus obtained from 30 s and 40 s samples, while the 65 s region used the average modulus between 60 s and 70 s. A Poisson’s ratio of 0.5 was assigned to both materials to reflect the incompressibility of the swollen hydrogel.

Swelling-induced deformation was simulated through thermal expansion, by assigning the experimentally determined linear swelling ratios as the materials’ coefficients of thermal expansion. A uniform temperature change of 1 °C was applied during the simulation step to trigger the equivalent swelling deformation.

The mesh was generated using hexahedral quadratic hybrid elements, with a global seed size of 0.25 mm. The NLGEOM option was activated to account for geometric nonlinearity due to large deformations.

To quantify the curvature induced by differential swelling, the coordinates of three nodes located on the outer surface of the bent construct (two at the lateral edges and one at the midpoint) were extracted. These coordinates were used to calculate the resulting curvature radius via geometric analysis.

## Figures and Tables

**Table 1:** Exposure time, gel fraction (Gel%), linear swelling (LS), polymer volume fraction in the reference state ($\phi_{0}$) and in the swollen state ($\phi_{s}$), and corresponding effective crosslinking density ($\nu_{e}$) estimated from the elastic modulus using rubber elasticity theory with phantom network correction.

Exposure Time [s]	E [kPa]	Gel% [%]	LS [%]	$\phi_{0}$ [%]	$\phi_{s}$ [%]	$\nu_{e}$ [mol m^−3^]
30	14.48	70.43	7.83	10.34	5.81	43.9
40	15.30	72.48	7.11	10.34	6.10	45.6
50	17.07	76.11	5.52	10.34	6.70	49.3
60	18.64	77.46	2.02	10.34	7.55	51.8
70	20.23	80.70	0.70	10.34	8.18	54.7

**Table 2:** Printing parameters used for 3D printing the hydrogel.

Parameter	Value
Layer height	50 μm
Bottom layers	0
Exposure time	20 to 70 s
Resting time after retraction	0.8 s
Lifting distance	3.0 + 1.4 mm
Lifting speed	15 + 55 mm/min
Retract distance	2.3 + 2.1 mm
Retract speed	145 + 45 mm/min

## Data Availability

The raw data supporting the conclusions of this article will be made available by the authors on request.

## Supplementary Materials

The following supporting information can be downloaded at: https://www.mdpi.com/article/10.3390/gels12050382/s1, Table S1: Central composite design (DoE) matrix with factor levels for GelMA, LAP, tartrazine concentration, and layer thickness; Table S2: DoE experimental matrix including measured transmittance (T), absorbance (A), and calculated attenuation coefficients (ε) for each formulation.
