# Supplementary material for: In Situ Programming of Shape-Morphing Hydrogels via Vat Photopolymerization for 4D Bioprinting
Source: Gels. 2026 Apr 30;12(5):382. doi: 10.3390/gels12050382 (PMC13205297; doi:10.3390/gels12050382)
Supplement: Supplementary file 1 [file gels-12-00382-s001.zip › gels-4265003-supplementary.pdf]

gels

In Situ Programming of Shape-Morphing Hydrogels via Vat Photopolymerization for 4D Bioprinting

Luca Guida[1,*], Elisa Ciotti[1], Giovanni Venturelli[1], Simone Bagatella[1] and Marinella Levi[1,*]

[1] Department of Chemistry, Materials and Chemical Engineering "Giulio Natta", Politecnico di Milano, Piazza Leonardo da Vinci 32, 20133, Milan, Italy
* Correspondence: (LG) luca.guida@polimi.it; (ML) marinella.levi@polimi.it

Supplementary information

Material formulation

The shape-morphing behavior investigated in this work is governed by differential swelling, which arises from spatial variations in crosslinking density within the same construct. Consequently, the formulation of the bioink must be carefully optimized to limit light penetration through previously cured layers, thereby preventing unintended over-crosslinking and preserving the programmed crosslinking gradients responsible for the morphing response.

To this end, a design of experiments (DoE) approach was employed to systematically identify the optimal bioink composition and to determine the most relevant factors affecting optical attenuation. A central composite design was implemented using JMP 18 (JMP Statistical Discovery LLC, Cary, NC, USA). Transmittance was selected as the response variable to be minimized, in order to reduce radiation penetration and maintain layer-by-layer control over crosslinking. The main effects and interactions of GelMA concentration (% w/v), LAP concentration (% w/v), tartrazine concentration (mM), and hydrogel layer thickness (µm) were investigated. The selected parameter ranges were defined based on prior literature, with GelMA concentration varying between 5 and 15% (w/v), LAP between 0.5 and 1.0% (w/v), tartrazine between 1 and 2 mM, and layer thickness between 200 and 300 µm [1–5]. The full set of experimental conditions is reported in **Table S1**.

The optical characterization was performed by measuring the emitted light intensity of the printer and the transmitted intensity through the bioink samples. Measurements were carried out using a SpectriLight ILT950 spectroradiometer (International Light Technologies Inc., Peabody, MA, USA) in a dark environment to avoid ambient light interference. For each formulation defined by the DoE, bioink samples were placed between two quartz plates with controlled spacing to ensure a defined thickness. The irradiance was obtained by integrating the spectral intensity over the wavelength range of 395–415 nm, corresponding to the nominal emission range of the 3D printer (405 nm). Transmittance was then calculated as the ratio between transmitted and incident irradiance.

Based on the experimental data, an effective attenuation coefficient was estimated using the Lambert-Beer law. This coefficient was then used to calculate the expected transmittance at a layer thickness of 50 µm, corresponding to the printing resolution, which could not be directly measured due to limitations in the dimensional accuracy of the optical setup. The bioink formulation was subsequently selected based on a target condition of achieving a transmittance ≤ 50% within a maximum of two layers. This threshold was defined as a practical design criterion to ensure sufficient attenuation while maintaining

 https://doi.org/10.3390/gels12050382

printability, and was further validated through printing and shape-morphing experiments.

Table S1. Central composite design (DoE) matrix with factor levels for GelMA, LAP, tartrazine concentration, and layer thickness.

Test	GelMA [% wt]	LAP [% wt]	Tartrazine [mM]	Thickness [μm]
1	5	0.5	1	200
2	5	0.5	1	300
3	5	0.5	2	200
4	5	0.5	2	300
5	5	0.75	1.5	250
6	5	1	1	200
7	5	1	1	300
8	5	1	2	200
9	5	1	2	300
10	10	0.5	1.5	250
11	10	0.75	1	250
12	10	0.75	1.5	200
13	10	0.75	1.5	250
14	10	0.75	1.5	300
15	10	0.75	2	250
16	10	1	1.5	250
17	15	0.5	1	200
18	15	0.5	1	300
19	15	0.5	2	200
20	15	0.5	2	300
21	15	0.75	1.5	250
22	15	1	1	200
23	15	1	1	300
24	15	1	2	200
25	15	1	2	300

The results of the DoE are reported in **Table S2**. Among the investigated factors, only GelMA concentration and tartrazine concentration exhibited a statistically significant

https://doi.org/10.3390/gels12050382

effect on transmittance ($p < 0.05$). However, inspection of the main effect plots indicated that tartrazine is the dominant factor governing light attenuation, while the contribution of GelMA is comparatively limited.

Table S2: DoE experimental matrix including measured transmittance (T), absorbance (A), and calculated attenuation coefficients (ε) for each formulation.

Test	GelMA [% wt]	LAP [% wt]	Tartrazine [mP]	Thickness [μm]	T [%]	A [ ]	ε [mM^{-1} mm^{-1}]
1	5	0.5	1	200	34.5	0.46	2.31
2	5	0.5	1	300	24.2	0.62	2.06
3	5	0.5	2	200	12.7	0.89	2.24
4	5	0.5	2	300	6.5	1.19	1.98
5	5	0.75	1.5	250	18.5	0.73	1.95
6	5	1	1	200	33.1	0.48	2.40
7	5	1	1	300	22.2	0.65	2.18
8	5	1	2	200	12.3	0.91	2.27
9	5	1	2	300	6.4	1.19	1.99
10	10	0.5	1.5	250	19.7	0.71	1.88
11	10	0.75	1	250	31.6	0.50	2.00
12	10	0.75	1.5	200	23.9	0.62	2.07
13	10	0.75	1.5	250	22.5	0.65	1.73
14	10	0.75	1.5	300	18.0	0.74	1.65
15	10	0.75	2	250	9.6	1.02	2.03
16	10	1	1.5	250	19.3	0.71	1.91
17	15	0.5	1	200	38.5	0.41	2.07
18	15	0.5	1	300	26.9	0.57	1.90
19	15	0.5	2	200	16.1	0.79	1.98
20	15	0.5	2	300	7.9	1.10	1.84
21	15	0.75	1.5	250	19.4	0.71	1.90
22	15	1	1	200	42.0	0.38	1.89
23	15	1	1	300	27.5	0.56	1.87
24	15	1	2	200	14.4	0.84	2.11
25	15	1	2	300	7.9	1.11	1.84

https://doi.org/10.3390/gels12050382

Based on this observation, the attenuation behavior was modeled considering tartrazine as the primary absorbing species. An effective attenuation coefficient was therefore calculated using the Lambert-Beer law across all experimental conditions, yielding a value of 2.00 mM^{-1} mm^{-1} with a relative standard deviation of ±8.94%. The relatively low variability supports the assumption that GelMA has a negligible contribution to light absorption within the investigated range.

The obtained coefficient was then used to estimate the transmittance at the characteristic printing scale. Given a layer thickness of 50 µm, and considering a maximum acceptable penetration over two consecutive layers (i.e., 100 µm total thickness), the transmittance was evaluated as a function of tartrazine concentration. Under these conditions, a minimum concentration of 1.5 mM was required to reduce the transmitted intensity below 50%.

Accordingly, this concentration was selected for the final formulation. GelMA and LAP concentrations were set at the upper bound of the investigated range (15% and 1% w/v, respectively) to promote rapid crosslinking and improve printing efficiency, compensating for the increased light attenuation induced by the absorber.

1. Ghosh, R.N.; Thomas, J.; B. R., V.; N. G., D.; Janardanan, A.; Namboothiri, P.K.; Peter, M. An Insight into Synthesis, Properties and Applications of Gelatin Methacryloyl Hydrogel for 3D Bioprinting. *Mater. Adv.* **2023**, *4*, 5496–5529, doi:10.1039/D3MA00715D.
2. Chalard, A.E.; Dixon, A.W.; Taberner, A.J.; Malmström, J. Visible-Light Stiffness Patterning of GelMA Hydrogels Towards In Vitro Scar Tissue Models. *Front. Cell Dev. Biol.* **2022**, *10*, doi:10.3389/fcell.2022.946754.
3. Nguyen, A.K.; Goering, P.L.; Reipa, V.; Narayan, R.J. Toxicity and Photosensitizing Assessment of Gelatin Methacryloyl-Based Hydrogels Photoinitiated with Lithium Phenyl-2,4,6-Trimethylbenzoylphosphinate in Human Primary Renal Proximal Tubule Epithelial Cells. *Biointerphases* **2019**, *14*, doi:10.1116/1.5095886.
4. Jeong, D.; Seo, J.W.; Lee, H.; Jung, W.K.; Park, Y.H.; Bae, H. Efficient Myogenic/Adipogenic Transdifferentiation of Bovine Fibroblasts in a 3D Bioprinting System for Steak-Type Cultured Meat Production. *Advanced Science* **2022**, *9*, doi:10.1002/advs.202202877.
5. Duong, V.T.; Lin, C. Digital Light Processing 3D Bioprinting of Gelatin-Norbornene Hydrogel for Enhanced Vascularization. *Macromol. Biosci.* **2023**, *23*, doi:10.1002/mabi.202300213.
